# The genetic basis of natural variation in mushroom body size in *Drosophila melanogaster*

**DOI:** 10.1038/ncomms10115

**Published:** 2015-12-11

**Authors:** Liesbeth Zwarts, Lies Vanden Broeck, Elisa Cappuyns, Julien F. Ayroles, Michael M. Magwire, Veerle Vulsteke, Jason Clements, Trudy F. C. Mackay, Patrick Callaerts

**Affiliations:** 1 KU Leuven—University of Leuven, Department of Human Genetics, Laboratory of Behavioral and Developmental Genetics, B-3000 Leuven, Belgium; 2 VIB Center for the Biology of Disease, B-3000 Leuven, Belgium; 3 Department of Biological Sciences, Program in Genetics and W. M. Keck Center for Behavioral Biology, North Carolina State University, Raleigh, North Carolina, 27695-7614, USA

## Abstract

Genetic variation in brain size may provide the basis for the evolution of the brain and complex behaviours. The genetic substrate and the selective pressures acting on brain size are poorly understood. Here we use the *Drosophila* Genetic Reference Panel to map polymorphic variants affecting natural variation in mushroom body morphology. We identify 139 genes and 39 transcription factors and confirm effects on development and adult plasticity. We show correlations between morphology and aggression, sleep and lifespan. We propose that natural variation in adult brain size is controlled by interaction of the environment with gene networks controlling development and plasticity.

## Abstract (web summary)

The mushroom bodies (MBs) in an insect brain integrate and process sensory information. Using fully sequenced/inbred lines of the Drosophila Genetic Reference Panel, this study performs genome wide association analyses and identifies candidate genes affecting MB size, and uses RNAi to functionally validate the identified loci.

## Graphical abstract

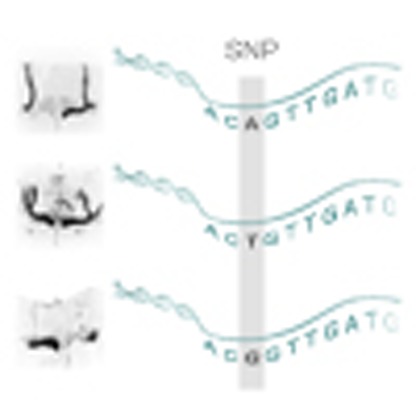

The brain plays a central role in controlling the social interactions of animals and their interactions with the environment. Visual, auditory, olfactory, tactile and gustatory sensory stimuli are integrated by the brain and result in a behavioural response appropriate for the context. Key higher order brain centres for integration and processing of sensory information are the mammalian cerebral cortex and the insect mushroom bodies (MBs). The size of the cerebral cortex and the MBs has been regarded as a proxy for cognitive ability and behavioural plasticity. For instance, the social brain hypothesis states that the evolution of the large brain size of primates is driven by the requirement to live in large groups^1^. By analogy, it was proposed that the large MBs in insects such as bees, wasps and ants are also driven by sociality^2^. However, the observation that solitary bee, wasp and ant species also have large MBs puts this notion in doubt and rather suggests that a common ancestor of these species had large MBs thereby providing the neural substrate on which sociality could evolve^2^,^3^. Other solitary insects with large MBs include cockroaches, herbivorous scarab beetles and some butterfly species^4^. Variation in brain size and MB size in particular is of great interest to understand the evolution of the brain and the mechanisms that govern this. The genetic substrate and the selective pressures acting on brain size as a quantitative trait are poorly defined.

Variation in brain size in the wild has been studied using two complementary approaches: interspecific comparative studies of the relationship between brain size and behavioural and environmental factors, and analyses of adaptive phenotypic plasticity^5^. Comparison of MBs in a broad spectrum of insects reveals big differences in size^6^,^7^. These size differences depend at least in part on significantly expanded neuroblast numbers. *Drosophila* MBs are derived from a total of eight MB neuroblasts, while *Apis* MBs are produced by four neuroblast clusters each consisting of 500 neuroblasts^2^,^8^. Thus, changes in developmental programmes are likely contributors to MB size differences. MB size in the adult insect also displays profound plasticity. Calyx volumes have been shown to be associated with age, sex and dominance behaviour in different paper wasp species^9^. In honeybees, experience modulates both dendritic spine morphology in the calyx and neuropil volume^6^,^10^. In ants, MB volumes vary between sexes and casts, and these volume changes are task dependent^11^,^12^. In *Drosophila*, MB fibre number has been related to age, sex and experience^13^,^14^.

The *Drosophila* MBs consist of ∼2,500 Kenyon cells projecting their axons rostroventrally through the peduncle, where at the MB heel they form distinct lobes^8^. All MB neurons are derived from eight MB neuroblasts and are subdivided in three groups: the α/β and α'/β' neurons with neurites that project to a medial and a vertical lobe, and the γ neurons that only project medially. The γ neurons arise during early larval stages and initially project both medially and vertically. The γ neurons are remodelled during pupal stages to form their characteristic medial MB lobe. The α'/β' and α/β neurons develop during late larval and pupal stages to form their respective lobes^8^.

In the current study, we use the inbred, sequenced lines of the *Drosophila* Genetic Reference Panel (DGRP)^15^,^16^ to demonstrate considerable natural genetic variation in length and width of the α and β MB lobes that is associated with variation in behavioural traits, and to perform genome wide association (GWA) analyses as a genetic screen to identify candidate genes affecting MB size. We identify the top candidate genes affecting MB size as those which have significantly associated genetic variants located within the gene and that are expressed in the MB neurons. In addition, we identify transcription factor-binding motifs that might be affected by variants in putative regulatory gene regions associated with MB size. We functionally validate the role of candidate genes and transcription factors in MB development and plasticity by knocking down their expression in the MBs during development and as adults using RNA interference (RNAi). We thus identify genes with known and unknown functions in MB development and plasticity; suggesting that the genes required to form these structures also cause naturally occurring quantitative genetic variation in size, and that GWA analyses of natural variation is an efficient gene discovery tool.

## Results

### Natural variation in mushroom body morphometry

We assessed morphology of the MB α and β lobes in 40 DGRP lines. These lobes are straightforward to visualize and we have previously optimized their morphometric analysis^17^,^18^,^19^. Animals of similar age and kept under constant environmental conditions were used throughout the study to minimize experience-dependent effects of mushroom body parameters (see Materials and Methods for more details).

**Figure 1:**
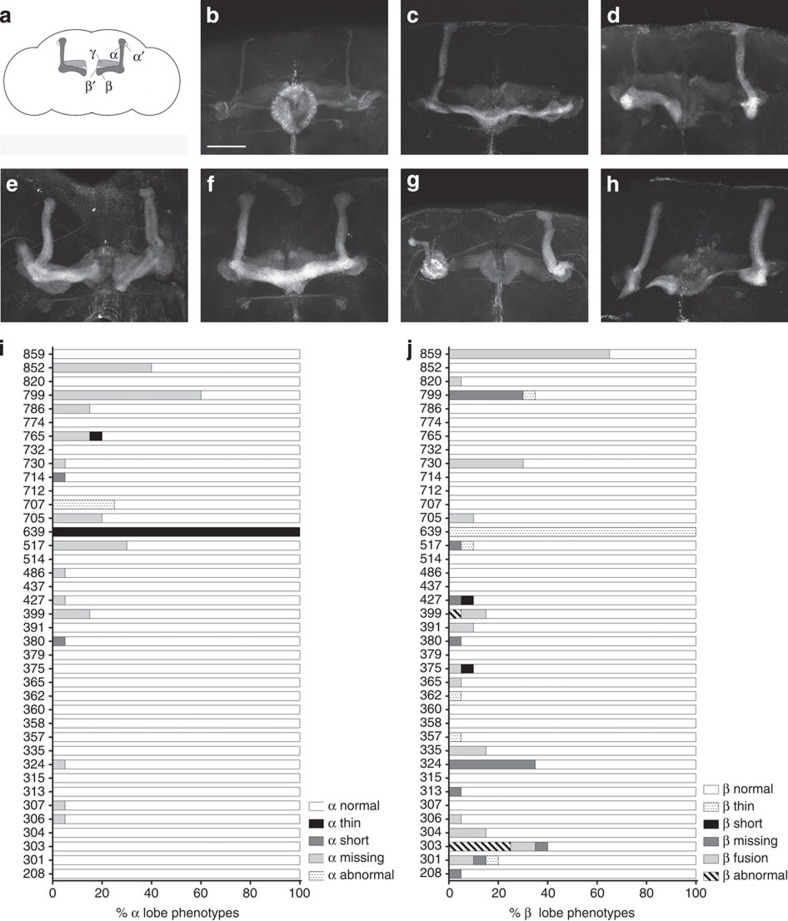
Variation in gross MB defects in the 40 DGRP lines. (**a**–**h**) Anti-FasII staining visualizing the αβ lobes of the MBs in the adult brain of 3–7 day old males of the 40 DGRP lines (Scale bar, 50 μm). (**a**) Scheme representing the different MB lobes in the adult brain (**b**) DGRP–639 males show thin αβ lobes. (**c**) DGRP–705 males show missing α lobes (**d**) DGRP–517 show thin α lobes and missing β lobes. (**e**) DGRP–707 males show defasciculation of αβ lobes and abnormal guidance of β lobes. (**f**) DGRP–730 males show β lobe fusion. (**g**) DGRP–799 males show missing β lobes and abnormal guidance and outgrowth of α lobes (**h**) DGRP–303 males show thin and abnormally formed β lobes. (**i**,**j**) Quantification of variation in gross MB defects in the 40 DGRP lines (*N*=20 hemispheres). (**i**) α lobe defects. (**j**) β lobe defects.

**Figure 2:**
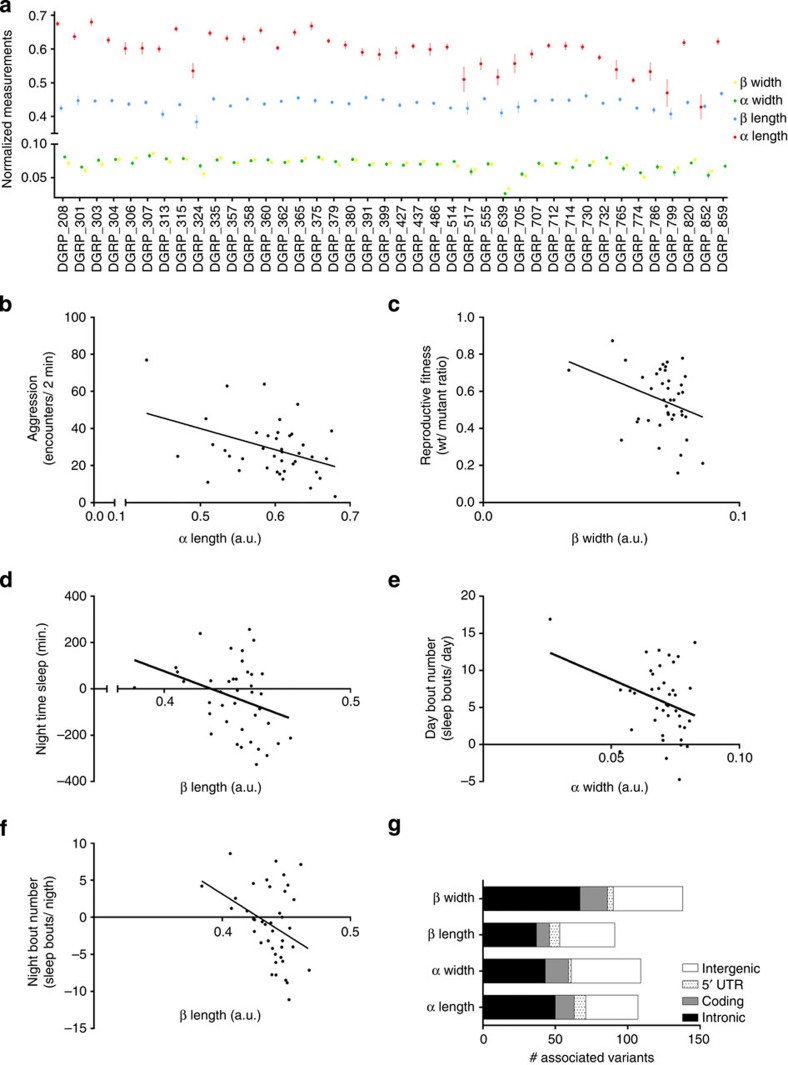
The 40 lines of the DGRP show variation in MB morphology correlated with different behaviours. (**a**) Observed variation in MB morphology traits in the 40 lines of the DGRP (*N*=20 hemispheres, Mean±s.e.m.). (**b**) α-lobe length is negatively correlated with aggression (Pearson's *r*=−0.410; *P*=0.009, *N*=20). (**c**) β-lobe width is negatively correlated with fitness (Pearson's *r*=−0.322; *P*=0.042, *N*=20). (**d**) β-lobe length is negatively correlated with night time sleep (Pearson's *r*=−0.323; *P*=0.044, *N*=20). (**e**) α-lobe width is negatively correlated with the number of sleep bouts during the day (Pearson's *r*=−0.315; *P*=0.047, *N*=20). (**f**) β-lobe length is negatively correlated with the number of sleep bouts during the night (Pearson's *r*=−0.361; *P*=0.023, *N*=20). (**g**) Genomic locations of variants associated with different MB morphology traits.

Surprisingly, we found gross morphological defects in 31 of the lines (77.5%), including thin lobes, short lobes, missing lobes, abnormal lobes (such as defasciculation and general malformation or misguidance) and fusion of β lobes. Some of the phenotypes were highly penetrant in certain lines, while others were seen more sporadically (Fig. 1; Supplementary Data 1). We attribute these gross morphological defects to fixation of recessive mutations affecting MB architecture. We quantified subtle variations in MB morphology by measuring the length and width of the α and β lobes^17^,^18^,^19^. All four traits were significantly variable, with broad sense heritabilities of respectively 0.274 and 0.376 for the length and width of the α lobes; and 0.123 and 0.313 for the length and width of the β lobes (Fig. 2a; Supplementary Data 2). All four traits are positively correlated with each other (Supplementary Data 3).

These DGRP lines have been extensively tested in various behavioural paradigms, enabling us to identify possible MB morphological correlates with behaviours. We assessed the correlations (*r*) for each MB parameter and aggressive behaviour, copulation latency, fitness, startle response, ethanol resistance, starvation resistance, lifespan, chill coma recovery and sleep traits^20^,^21^,^22^. All behavioural data were male specific except for mating and fitness traits, for which both sexes were pooled. We found significant negative correlations between aggression and α lobe length (*r*=−0.410, *P*=0.009), β lobe width and fitness (*r*=−0.322, *P*=0.042), β lobe length and night time sleep (*r*=−0.323, *P*=0.044), α lobe width with the number of sleep bouts during the day (*r*=−0.315, *P*=0.047) and β lobe length with the number of sleep bouts during the night (*r*=−0.361, *P*=0.023; Fig. 2b–f; Supplementary Data 4). Although MBs have been shown to be involved in the regulation of aggression and sleep, this is, to our knowledge, the first demonstration that natural variation in MB morphology is associated with these traits^19^,^23^,^24^,^25^,^26^.

### GWA analyses of mushroom body morphological variation

We performed single marker GWA analyses for the four genetically variable traits (length and width of the α and β lobes). At a reporting *P* value threshold of *P*<10^−5^, we identified variants in annotated genes, as well as variants in intergenic regions (not located in an annotated gene) associated with MB morphology^27^. The former variants implicate candidate genes affecting MB size, while the latter could potentially contain regulatory motifs indicative of transcription factor-binding sites regulating these traits. We identified 104 variants associated with α lobe length, 109 variants associated with α lobe width, 90 variants associated with β lobe length and 138 variants associated with β lobe width (Supplementary Data 5). On average, 62% of the variants were associated with genes (variants in the coding sequence, introns and the 3′ and 5′ untranslated region (UTR)); the remainder was intergenic (variants outside the transcribed region of an annotated gene) (Fig. 2g).

We mapped 39 candidate genes associated with α lobe length, 37 with α lobe width, 35 with β lobe length and 54 with β lobe width. These comprised 139 unique genes, as 18 genes were associated with two MB traits and four were associated with three mushroom body traits (Supplementary Data 6). Six of the candidate genes are known to regulate MB development (starry night (*stan*), frizzled (*fz*), Protostome-specific GEF (*PsGEF*), Protein tyrosine phosphatase 10D (*Ptp10D*), misshapen (*msn*), vein (*vn*)), four act in pathways that modulate MB development but have themselves never been linked to MB development (APP-like protein interacting protein 1 (*Aplip1*), Ecdysone-induced protein 63E (*Eip63E*), Ecdysone-induced protein 75B (*Eip75B*), *JIL-1*) and two are involved in adult MB function (slowpoke (*slo*), pumilio (*pum*))^28^,^29^,^30^,^31^,^32^,^33^,^34^,^35^,^36^,^37^. No role in MB development or function has been reported for the remaining 127 genes. We performed gene ontology enrichment analyses to place the candidate genes into biological context^38^. Individual gene ontology categories and clusters were enriched for genes involved in development, morphogenesis, metamorphosis and behaviour (Supplementary Data 7 and 8).

**Figure 3:**
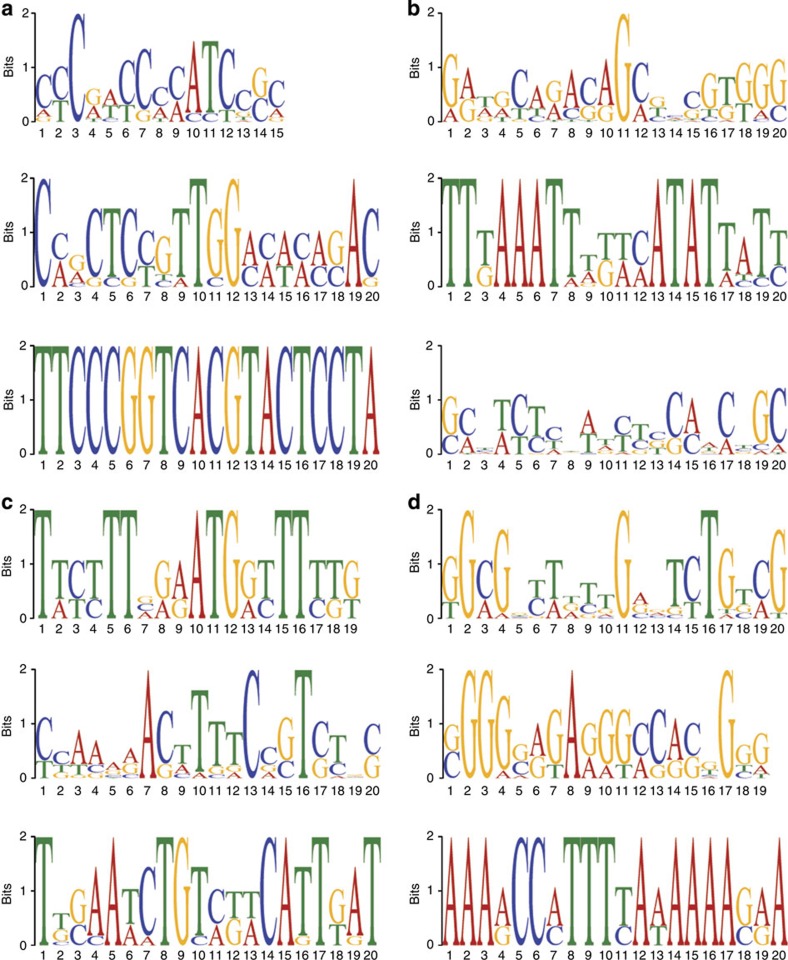
Recurring transcription factor-binding motifs in non-coding sequences with variants associated with MB parameters. (**a**) Motifs associated with α lobe length (motif 1: *P*=6,9 E-014; motif 2 *P*=5,4 E-010; motif 3: *P*=4,9 E-002). (**b**) Motifs associated with α lobe width (motif 1: *P*=2,6 E-015; motif 2 *P*=3,6 E-006; motif 3: *P*=2,5 E-012). (**c**) Motifs associated with β lobe length (motif 1: *P*=2,0 E-018; motif 2 *P*=3,2 E-010; motif 3: *P*=3,2 E-009). (**d**) Motifs associated with β lobe width (motif 1: *P*=4,1 E-014; motif 2 *P*=7,9 E-013; motif 3: *P*=1,4 E-005)

To assess whether variants in non-coding regions that are associated with MB size could be in regulatory regions, we used the Multiple EM for Motif Elicitation tool to identify recurring motifs for these variants for each of the genetically variable traits^39^. We selected the three most significantly recurring motifs for each trait (Fig. 3). We then used the TOMTOM motif comparison tool to identify the transcription factor(s) most likely to bind to these motifs^40^, and categorized them based on their patterns of association with MB traits (all four traits, α and β lobe length, α and β lobe width, α lobe width and length, β width and length) (Supplementary Data 9). Of these 39 candidate transcription factors, longitudinals lacking (*lola*), tailless (*tll*) and ftz transcription factor 1 (*ftz-f1*) have been previously shown to be involved in MB development or function^17^,^41^,^42^. Dissatisfaction *(dsf)* and lethal of scute (*l(1)sc)* are expressed in the MBs^43^,^44^. Abdominal B (*Abd-B)* and hunchback (*hb)* interact with pathways with a previously described function in MB development or function^45^,^46^. Many of the other transcription factors are involved in (neuro-) developmental processes, but have no known role in the MBs.

In summary, we identified 139 candidate genes and 39 candidate transcription factors associated with morphological variation in MB lobes. Although many of these genes are involved in development, morphogenesis and metamorphosis, only eight genes and four transcription factors have been previously shown to act in the MBs.

### Functional validation analyses

We hypothesized that the most promising candidates for functional validation would be candidate genes that are expressed in the MBs. Therefore, we used the FlyLight database to restrict the number of candidate genes for functional validation tests to those with MB expression^47^. A total of 264 lines, containing enhancer sequences of 26 genes of the total 139 candidate genes, were present in the FlyLight database; of these, 44 drove expression in the MBs. Note that this may deviate from the actual number as the FlyLight lines may not always be a complete representation of the real expression pattern (own unpublished observations for the *eyeless* gene). In total, we found a total of 20 unique genes which showed expression in the MBs. We included *pum* as a candidate gene for functional validation because it is known to be expressed in the MB and play a role in synaptic plasticity in the adult brain, although a FlyLight line was not available^37^. We also used the FlyLight database to determine MB expression for the candidate transcription factors, of which 44 lines, containing enhancer sequences of 21 genes of the total 39 candidate transcription factors, were present in the FlyLight database (Supplementary Data 10). Thirty-two of these lines showed MB expression. In total these represented 17 unique transcription factors. We verified the MB expression of all lines by driving green fluorescent protein (GFP)–CD8 expression with the different GAL4 lines followed by anti-GFP immunostaining (Supplementary Data 10), and confirmed all but one (*acj6*). In addition, we determined that all enhancer sequences in the FlyLight lines corresponded to the reported gene by means of PCR (Supplementary Data 10). In summary, we confirmed MB expression of 20 candidate genes and 16 candidate transcription factors associated with one or more MB traits, the large majority of which are novel with respect to a role in MB function.

**Figure 4:**
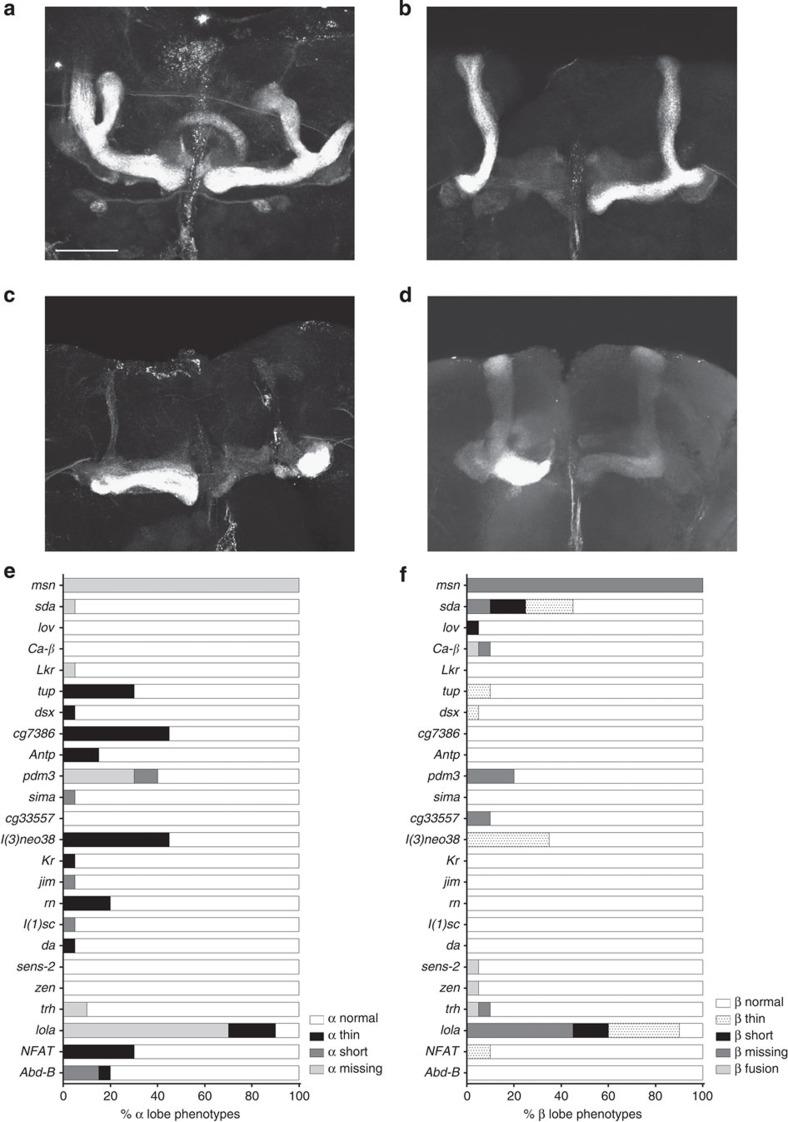
RNAi-mediated knockdown of the identified genes and transcription factors results in gross MB defects. (**a**–**d**) Anti-FasII staining visualizing the αβ lobes of the MBs in the adult brain of 3–7 day old males (scale bar, 50 μm) (**a**) *UAS-RNAi-AbdB*^*TRIP35647*^, *OK107-Gal4* (**b**) *UAS-RNAi-CG33557*^*VDRC23517*^, *OK107-Gal4* (**c**) *UAS-RNAi-lola*^*TRIP35721*^, *OK107-Gal4* (**d**) *UAS-RNAi-sda*^*TRIP 37494*^, *OK107-Gal4.* (**e**,**f**) Quantification of variation in gross MB defects on RNAi-mediated knockdown (*N*=20 hemispheres). (**e**) α lobe defects. (**f**) β lobe defects.

**Figure 5:**
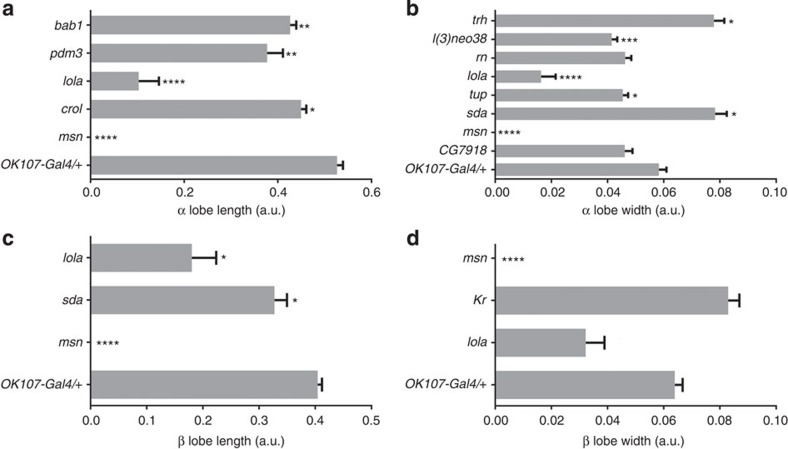
Significant alterations in MB morphology traits upon RNAi-mediated knockdown of the identified genes during development. (**a**) α lobe length. (**b**) α lobe width. (**c**) β lobe length. (**d**) β lobe width. (Kruskall–Wallis tests followed by Dunn's *post hoc* tests; *P<0.05; **P<0.01; ***P<0.001; ****P<0.0001; *N*=20, Mean±s.e.m.).

We used targeted RNAi knockdown in the MBs using the *OK107-Gal4* driver to validate the role of the candidate genes and transcription factors in MB morphology (Supplementary Data 10). This driver is expressed in all MB neuroblasts, ganglion mother cells and neurons from embryonic stages onwards^48^. For the candidate genes we focused on the genes that are expressed in the MBs themselves, for the transcription factors we analysed all 39 genes. We tested one RNAi line for each of the 60 genes. *OK107-Gal4* driven knockdown of *Eip75B, Mrtf* and *ftz-f1* resulted in lethality, therefore these genes could not be evaluated for MB phenotypes. For the remaining genes, we evaluated gross as well as subtle MB phenotypes of the RNAi knockdown genotypes compared to the control. No control animals had gross MB abnormalities, but 24 of the 57 remaining genes tested showed gross defects in MB morphology. These defects included thin and missing α lobes and thin, short, fused and missing β lobes (Fig. 4; Supplementary Data 11). A total of 10 genes had more subtle, quantitative effects on MB size. misshapen (*msn*) affected all four traits; *lola* affected α lobe length and width and β lobe length, and slamdance (*sda*) affected α lobe width and β lobe length. crooked legs (*crol*), pou domain motif 3 (*pdm3*) and bric a brac 1 (*bab1*) specifically affected α lobe length and tailup (*tup*), jim, lethal (3) neo8 (*l(3)neo8*) and trachealess (*trh*) specifically affected α lobe width (Fig. 5).

**Figure 6:**
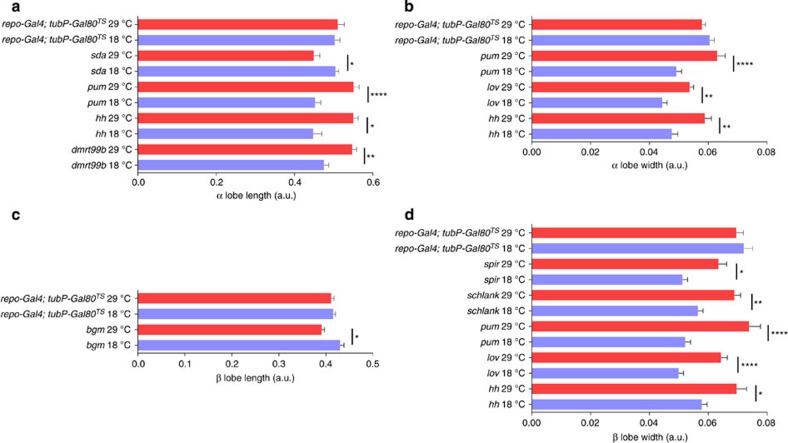
Significant alterations in MB morphology traits on RNAi-mediated knockdown of the identified genes in the adult MBs. (**a**) α lobe length. (**b**) α lobe width. (**c**) β lobe length. (**d**) β lobe width. (Kruskall–Wallis tests followed by Mann–Whitney tests with a Bonferroni correction; *P<0.05; **P<0.01; ***P<0.001; ****P<0.0001, *N*=20, Mean±s.e.m.) Red bars represent flies switched to 29 °C after eclosion and 4 days before dissection, blue bars represent control flies kept at 18 °C.

In many insect species, the MB is a dynamic structure in the adult brain, able to change in an experience-dependent manner^6^,^9^,^13^,^14^. Thus, it is possible that the candidate genes and transcription factors do not influence MB development *per se*, but rather adult plasticity. Support for this hypothesis comes from our observation that we identified *pum*, which is known for its role in plasticity in the adult MBs, as a candidate gene^37^. We could not include *pum* in the expression analysis since no FlyLight lines were available, but we did include it in our plasticity analysis as a positive control. To determine whether the candidate genes act in adult MB plasticity, we crossed RNAi lines targeting these genes with *OK107-Gal4* in combination with a *TubP-Gal80*^*TS*^ transgene to restrict the RNAi-mediated knockdown to adult stages by elevating the temperature to 29 °C. We only performed these analyses for the candidate genes. We identified eight genes that modulate adult MB plasticity (Fig. 6). *pum* and hedgehog (*hh*) affect plasticity in α lobe length and width and β lobe width and jim lovell (*lov*) affects plasticity of α and β lobe width. The other genes have specific effects on plasticity: slamdance (*sda)* and doublesex-Mab related 99B (*dmrt99B*) for α lobe length; bubblegum (*bgm*) for β lobe length; and spire (*spir*) and *schlank* for β lobe width.

## Discussion

Analysis of the genomic architecture of the DGRP panel highlighted extensive natural variation in the genomes of these flies^16^. These natural variations have been associated with multiple behaviours^20^,^49^,^50^. Furthermore, natural variation in volumes of brain regions, as well as in neuron numbers has been observed in humans and mice^51^,^52^. However, this is the first (to our knowledge) systematic analysis of natural variation in brain structures. We show that the DGRP exhibits variation in MB morphology with broad sense heritabilities ranging from 12 to 38%, which correlates with behavioural variation. In many lines, this variation is characterized by profound morphological differences. In humans and primates, overall brain size has been reported to be highly heritable, however, the reported heritability of the size of different substructures ranges from <5% to >80% (refs 53, 54).

Our GWA analyses identified many variants that are associated with variation in MB morphology, allowing us to identify genes and putative transcription factors involved in MB development. Of the 139 unique candidate genes, only eight have been previously reported to be involved in MB morphology or function^28^,^29^,^30^,^32^,^33^,^34^,^35^,^37^. Furthermore, more than one-third of the polymorphisms associated with variation in MB morphology were located in intergenic regions. We hypothesized that these intergenic regions, as well as other non-coding regions, would contain binding sites for transcription factors regulating MB morphology. Analysis of recurring motifs allowed us to select 39 putative binding transcription factors. Four of these had a known role in the MBs, including *lola* which was linked to all four MB traits, providing a proof of concept for our approach^17^; the other transcription factors were not known to affect MB morphology. We showed that the large majority of identified genes and transcription factors for which FlyLight lines are available indeed show expression in the MBs. Furthermore, we validated a functional role for these genes in MB development. Knockdown of many of the identified genes resulted in variation in MB morphology, ranging from sporadic gross defects to more subtle variations. The observed phenotypes may, however, be an underestimate as some RNAi constructs may not be sufficiently efficient to produce prominent phenotypes. At the same time, some false positives due to off-target effects cannot be excluded. Overall, the prominent MB expression of the identified genes and transcription factors and their causal effects reveal the biological relevance of our analysis and highlight the strength of GWA analyses of natural variation as an efficient gene discovery tool.

In addition to our analysis of the developmental roles of the candidate genes in the MBs, we performed the first systematic study of the genetic basis of variation in adult brain morphometry. We showed that our approach is valid to identify genes involved in structural plasticity in the MB. We investigated 22 genes, of which eight showed significant effects on adult MB morphology. Interestingly, *pum*, which is known for its role in behavioural and synaptic plasticity and is expressed in the MBs, had large effects^37^. We conclude that *pum* is also required for adult MB structural plasticity.

In mice, different brain regions are not constrained by developmental programmes and can thus evolve independently of other regions or overall brain size^55^. We focus on branches within one neuropil that derive from one neuron. The different MB traits are correlated, suggesting that these branches do not develop completely independently of each other. However, different variants or knockdown of different genes can have α or β lobe-specific effects, arguing for at least partially independent development within one neuropil. Branch-specific effects have been previously reported for *RhoGAPp190* (ref. 56). This protein is important for dorsal branch stability both during development and in the adult brain. Interestingly, this protein and two of its interactors, Integrin and Src, have both been implicated in learning and memory^56^. Hence, it was hypothesized that RhoGap signalling could play a role in adult brain plasticity underlying behavioural changes^56^. Of note, two of the genes we identified, *psGEF* and *CG30440*, are involved in Rho signalling.

Increases in brain volumes, including cortex and hippocampus, have been associated with enriched environments and learning in many species^6^. MBs are known to play a prominent role in multiple behaviours. We show that morphometric variations in this neuropil correlate with changes in aggression, fitness and sleep, implicating natural variation in MB morphology in behavioural differences. It remains to be determined whether the morphological differences are causally linked to the variation in behaviour. Complex behaviours and MB morphology are known to be influenced by pleiotropic genes affecting distinct processes, and thus the two sets of observations could be independent consequences of the action of pleiotropic genes^18^,^19^,^21^. However, it is equally possible that the observed changes in MBs represent a direct physical correlate that forms the basis of behavioural alterations. Lobe-specific morphological differences have been shown to underlie different aspects of memory formation^57^. Our current work confirms a previously reported correlation between the length of the MB α lobe and aggressive behaviour^18^, thereby demonstrating that this relation is robust and independent of genetic background. The relationship between MB structure and aggression is further supported by the fact that 19 of the identified genes and transcription factors are also candidate genes for aggressive behaviour^18^,^26^. MB size has also been shown to be correlated with aggressive behaviour in wasps, suggesting a conservation of the role of MB in aggression across species^9^. The MBs have previously been implicated in the regulation of sleep and many other behaviours important for survival, such as olfaction, interpretation of visual input and learning and memory^23^,^58^,^59^. We find correlations of MB structure with sleep and fitness. Interestingly, many of the variants that we identified have been reported to be associated with both sleep and fitness traits in the DGRP (22 with sleep, 12 with fitness traits)^20^,^60^. We propose that the changes in structure reflect changes in MB function affecting these traits. Interestingly, sleep and fitness traits have also been associated with alterations in brain size in humans^61^.

Changes in adult MBs have been observed in many insects. The mechanisms underlying these alterations are unknown. In certain insects, including crickets, neurogenesis of kenyon cells in the adult brain has been shown. However, in *Drosophila* and honeybees, this process is absent and can thus not explain the observed plasticity in MB morphometry^6^,^62^. Alterations in MB lobe morphometry are reminiscent of metamorphosis in both honeybees and *Drosophila*. During metamorphosis, MBs are remodelled through MB fibre outgrowth and regression independent of cell body proliferation or death^13^. We propose that a similar process of fibre shedding and regrowth could form the basis of MB volume changes during adulthood. Previously, it has been hypothesized that such processes form the mechanistic basis of plasticity in odour templates in the MB^63^,^64^. However, many other processes could be involved in morphometric or volumetric changes in adult MB. These include changes in the size and number of terminal branches, swelling of the involved axons or axonal branches, as well as spine formation and retraction^13^,^65^. In addition, non-neuronal alterations, such as swelling of glia, could be contributing factors. Our data provide a first insight into the genetic mechanisms underlying structural plasticity in the adult MB. We identified genes with a variety of different functions, suggesting a complex interplay between different processes influencing MB morphology. Identification of *spir*, an actin nucleation protein involved in cytoskeleton organization, and *hh*, shown to modulate adult brain regeneration, can argue for remodelling due to fibre outgrowth^66^. On the other hand, identification of genes involved in synaptic plasticity, such as *pum*, can argue that volumetric change can be due to local remodelling of synapses on present axons^37^.

We report the first systematic analysis of the genetic basis of natural variation in brain structures in *Drosophila*. Our approach offers an unbiased identification of candidate genes involved in both development and adult plasticity. We provide insights in the genetic mechanisms underlying these variations, as well as in the biological relevance of these morphological changes in the light of behavioural alterations. The overall genetic architecture of variation in brain structure and other complex traits in *Drosophila* is very similar to high resolution GWAS in humans and involves a large number of loci with relatively small effect size. Furthermore, epistasis and pleiotropy have also been demonstrated to be conserved features of the genes involved in complex traits^67^. However, our results are different from the situation in humans where ‘missing heritability' is frequently seen in studies of complex phenotypic traits^68^. We suspect that the main differences between our study and GWAS in human populations are that (1) we have used full sequence data; (2) we have stringently controlled the environment; (3) we have measured multiple individuals per genotype, thus improving the accuracy in estimating the true genetic value of each line; and (4) under a strictly additive model, the genetic variance of a population of inbred lines is twice that of the outbred population from which they were derived.

## Methods

### Fly stocks and husbandry

Flies were reared on standard cornmeal-agar-molasses *Drosophila* medium with a 12:12 light-dark cycle. For each cross, we used four to five virgin females and two males to obtain comparable levels of offspring density. Flies without *tubP-Gal80*^*TS*^ were maintained at 25 °C. Flies with the *tubP-Gal80*^*TS*^ allele were kept at 18 °C during development and switched to 29 °C 1–3 days after eclosion and 4 days before dissecting. All flies were between 3 and 7 days old at the time of dissection. We used the first 40 DGRP lines for which full sequencing data were available. Stocks from the Janelia Farm FlyLight Project, the TRIP collection, *OK107-Gal4* and *tubP-Gal80*^*TS*^ were obtained from the Bloomington stock center (Bloomington, IN, USA)^69^. KK and GD RNAi lines and their respective co-isogenic controls were ordered from the Vienna *Drosophila* Resource Center (Vienna, Austria).

### Immunohistochemistry

Adult brains from male flies were dissected and processed for immunohistochemistry. Mouse monoclonal anti-fasciclin 2 antibody (1:200; Developmental Studies Hybridoma Bank, University of Iowa, IO, USA) was used to visualize MB α and β lobes^17^,^18^,^19^,^26^. Anti-GFP (1:500) was obtained from Abcam, Cambridge, USA. Immunostainings were documented with an Olympus BX61 epifluorescence microscope equipped with a DP70 digital camera. Confocal imaging was performed using an Olympus FV1000 confocal microscope. Since the possibility existed that fasII expression levels themselves differ between DGRP lines (for example, due to effects of SNPs on fasII regulation) we adjusted fluorescence intensities whenever needed so that unambiguous measurements could be made. Overall, we never observed lobe-specific changes in FasII expression levels or any other difference that could impair accurately measuring mushroom body lobe parameters.

### Morphometric measurements

Length and width of the α and β lobes of the MBs were measured by using the analySIS FIVE software and expressed as values relative to the distance between the α lobe heels^17^,^18^,^19^. This internal calibration controls for differences in brain size when assessing variation in morphometric parameters among genotypes. Values were obtained for 10 brains for all genotypes, thus allowing analysis of 20 hemispheres.

### Quantitative genetic analyses

We partitioned the variation in the length and width of the α and β MB lobes using random effects factorial analysis of variance models of form Y=*L*+*H*+*L* × *H*+*ɛ*, where *L* denotes DGRP line, *H* is hemisphere and *ɛ* the within line variance. We computed the variance components (*σ*^2^) for each of these terms using restricted maximum likelihood and estimated broad sense heritabilities (*H*^2^) as *H*^*2*^=(*σ*_*L*_^2^+*σ*_*L × H*_^2^)/(*σ*_*L*_^2^+*σ*_*L × H*_^2^+*σ*_*ɛ*_^2^).

### GWA analyses of mushroom body size

We associated the line mean of each of the four MB traits with all segregating sites in the DGRP. We used the analysis of variance model *Y*=*μ*+SNP+ *ɛ* to evaluate each segregating site^15^, where *Y* is the phenotype, *μ* is the overall mean, SNP is the genotype and *ɛ* is the variance among line means within each genotype class. We used a nominal *P* value<10^−5^ as the reporting threshold for nominating candidate genes for functional validation. We annotated the site class of each significant variant as genic (variants in coding sequences, introns and UTR) or non-coding (variants outside the transcribed region of an annotated gene, UTR and introns)^27^.

### Identification of putative transcription factors

We inferred possible regulatory functions of variants associated with MB size located in non-coding regions. We analysed 40 basepairs up- and down-stream of each of these variants as this size is likely to span possible transcription factor-binding sites^70^. We entered these sequences in the Multiple EM for Motif Elicitation tool (version 4.9.0), separately for each MB trait, to identify shared motifs associated with each trait^39^. We selected the three most significant motifs (*P*<0.05) with an occurrence of zero or one per sequence. We then used the TOMTOM Motif Comparison Tool to compare the significant motifs with a database of known *Drosophila* motifs and to predict putative transcription factors (*P*<0.05) binding to these motifs^40^. We then classified the transcription factors based on their association with MB traits: all four traits, β and α lobe length, β and α lobe width, α lobe width and length, β lobe width and length. Finally, we selected the most significant predicted transcription factors from each group for further analysis.

### Expression and functional analyses

We used the FlyLight database^69^ to analyze the expression patterns of the candidate genes and transcription factors. We checked the availability of reporter lines in the FlyLight database. We included all transcription factors in this analysis. For genic variants we selected genes expressed in the MB for further analyses. We verified all FlyLight line inserts using PCR. The forward primer was vector specific (5′-AAATAGGGGTTCCGCGCACAT-3′) and reverse primers were made against the relevant fragments for each line (Supplementary Data 12). We verified MB expression of all FlyLight candidate genes described as showing MB expression, as well as for all available candidate transcription factor lines using immunohistochemistry (anti-GFP).

We performed MB specific, RNAi-mediated knockdown of the selected genes and transcription factors using *OK107-Gal4* (Supplementary Data 10). For experiments without *tubP-Gal80*^*TS,*^, dissected males were between 4 and 7 days old and kept at 25 °C. *OK107-Gal4* was crossed to the RNAi empty vector progenitor strain to obtain heterozygous control males. For experiments with *tubP-Gal80*^*TS*^, males were kept at 18 °C during development and switched to 29 °C after 1–3 days after eclosion and 4 days before dissection. Control males were kept at 18 °C throughout. For each condition, we analysed 20 hemispheres. The effect of RNAi-mediated knockdown on MB parameters during development was statistically analysed using non-parametric Kruskall–Wallis tests followed by Dunn's *post hoc* tests. All lines were compared with the heterozygous *y*^*1*^*,v*^*1*^*;OK107-Gal4* line. The effects of RNAi-mediated knockdown on MB parameters in the adult brain were statistically analysed using non-parametric Kruskall–Wallis tests followed by Mann–Whitney tests with a Bonferroni correction. Each genotype at 29 °C was compared with the same genotype at 18 °C. To control for possible effects due to the temperature shift, we used heterozygous *y*^*1*^*,v*^*1*^*;OK107-Gal4; tubP-Gal80*^*TS*^ flies at 18 °C and shifted to 29 °C as a control.

## Additional information

**How to cite this article:** Zwarts, L. *et al.* The genetic basis of natural variation in mushroom body size in *Drosophila melanogaster*. *Nat. Commun.* 6:10115 doi: 10.1038/ncomms10115 (2015).

## Supplementary Material

Supplementary Data 1 Percentage of gross MB phenotypes observed in the 40 DGRP lines

Supplementary Data 2 ANOVAs of mushroom body traits.

Supplementary Data 3 Correlation between the analyzed MB traits in the 40 DGRP lines (Pearson's correlation coefficient)

Supplementary Data 4 Correlation between the analyzed MB traits and different behaviors in the 40 DGRP lines (Pearson's correlation coefficient)

Supplementary Data 5 GWA analyses of mushroom body morphological variation

Supplementary Data 6 Genes with variants associated with multiple MB traits

Supplementary Data 7 Enriched gene ontology categories among genes with variants associated with MB morphology traits

Supplementary Data 8 Cluster analyses of gene ontology terms

Supplementary Data 9 Selected transcription factors

Supplementary Data 10 used FlyLight, TRIP and VDRC lines

Supplementary Data 11 Percentage of gross MB phenotypes observed upon RNAi mediated knock down of the investigated genes

Supplementary Data 12 Primers used for the verification of used FlyLight lines
